# Music Emotion Detection Using Hierarchical Sparse Kernel Machines

**DOI:** 10.1155/2014/270378

**Published:** 2014-03-03

**Authors:** Yu-Hao Chin, Chang-Hong Lin, Ernestasia Siahaan, Jia-Ching Wang

**Affiliations:** Department of Computer Science and Information Engineering, National Central University, Taoyuan 32001, Taiwan

## Abstract

For music emotion detection, this paper presents a music emotion verification system based on hierarchical sparse kernel machines. With the proposed system, we intend to verify if a music clip possesses happiness emotion or not. There are two levels in the hierarchical sparse kernel machines. In the first level, a set of acoustical features are extracted, and principle component analysis (PCA) is implemented to reduce the dimension. The acoustical features are utilized to generate the first-level decision vector, which is a vector with each element being a significant value of an emotion. The significant values of eight main emotional classes are utilized in this paper. To calculate the significant value of an emotion, we construct its 2-class SVM with calm emotion as the global (non-target) side of the SVM. The probability distributions of the adopted acoustical features are calculated and the probability product kernel is applied in the first-level SVMs to obtain first-level decision vector feature. In the second level of the hierarchical system, we merely construct a 2-class relevance vector machine (RVM) with happiness as the target side and other emotions as the background side of the RVM. The first-level decision vector is used as the feature with conventional radial basis function kernel. The happiness verification threshold is built on the probability value. In the experimental results, the detection error tradeoff (DET) curve shows that the proposed system has a good performance on verifying if a music clip reveals happiness emotion.

## 1. Introduction

Listening to music plays an important role in human's daily life and people usually gain much benefit from listening to music. Besides the leisure purpose, music listening has other application areas such as education, inspiration production, therapy, and marketing [1]. Sometimes people try to be in particular emotion state by listening to music. However, in such situation, people need to choose the music which can make human have particular feelings. They should listen to each song at least once to know the music emotion of each song, and the whole process takes much time. If people can use computer to detect the emotion content in music, the problem can be solved. Besides this application, music emotion detection technology can be applied to other area as well, such as music research, music recommendation, and music retrieval. For the limitless potential of music emotion detection technology, many researchers focus on detecting emotion in music.

Many researches on music emotion detection have been proposed in music emotion detection [2]. Existing research methods could be divided into two main categories: dimension approach and categorical approach. Dimension approach defines an emotion plane and views the emotion plane as a continuous emotion state space. Each position of the plane means an emotion state [3]. The acoustical features can be mapped to a point in the emotion plane [4]. Categorical approach works by categorized emotions into a number of emotion classes. Each emotion class represents an area in the emotion plane [3]. Different from dimension approach, each emotion class is defined clearly. In the training phase, acoustical features are directly used to train classifiers to recognize the corresponding emotion classes [5]. In this paper, the proposed method belongs to the second type.

In previous music emotion detection studies, many machine learning algorithms are applied. In [5], features were mapped into emotion categories on the emotion plane, and two support vector regressors were trained to predict the arousal and valence value. In [6], hierarchical framework was adopted to detect emotion from acoustic music data. The method has the advantage of emphasizing proper feature in different detection work. In [7], support vector machine was applied to detect emotion content in music. In [8], kernel-based class separability is used to weight features. After feature selection, principal component analysis and linear discriminant analysis were applied, and *k*-nearest neighborhood (KNN) classifier was then implemented. In this paper, a music emotion detection system is proposed. The system establishes a hierarchical sparse kernel machine. In the first level, eight 2-class SVM models are trained, with eight emotion classes as the target sides, respectively. It is noted that emotion perception is usually not based on a single acoustical feature but a combination of acoustical features [4, 9]. This paper adopts an acoustical feature set comprising root mean square energy (RMS energy), tempo, chromagram, MFCCs, spectrum centroid, spectrum spread, and ratio of a spectral flatness measure to a spectral center (RSS). Each of them is normalized. In the second level of hierarchical sparse kernel machines, a 2-class relevance vector machine (RVM) model with happiness as the target side and other emotion as the background side is trained. Besides, first-level decision vector is used as the feature in this level.

The rest of this paper is organized as follows. The system overview is described in Section 2. The features and first-level decision vector extraction are described in Section 3. Principle component analysis is described in Section 4. The introduction of SVM and RVM is described in Section 5.  Section 6 shows our experimental results. The conclusion is given in Section 7.

## 2. System Overview

**Figure 1:**
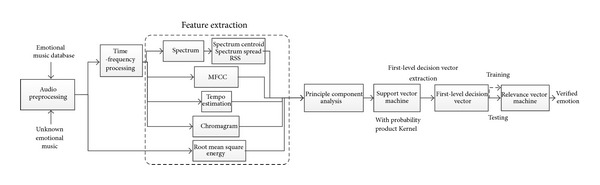
Block diagram of the proposed system.

The block diagram of the proposed system is presented in Figure 1. The system mainly comprises two level sparse kernel machines. For the first-level SVMs, we use a set of acoustical features which includes RMS energy, tempo, chromagram, MFCCs, spectrum centroid, spectrum spread, and RSS. In Table 1, the used acoustical features are classified into four main types, that is, dynamic, rhythm, timbre, and tonality. Because each feature's scale is different, normalization of the whole feature set is performed [10]. After normalization, eight SVM models are trained to transform acoustical features into emotion profile features. Each of the eight SVM model is trained and tested using probability product kernel. We use the first-level decision vectors generated from the angry, happy, sad, relaxed, pleased, bored, nervous, and peaceful emotion classes. For an emotion, to calculate the corresponding value in the emotion profile features, we construct its 2-class SVM with calm emotion as the background side of the RVM. For the RVM, conventional radial basis function kernel is used, and the first-level decision vector extracted in the first level is utilized as the feature. To verify happiness emotion, a 2-class RVM with happiness as the target side and other emotion as the background side is constructed. For a tested music clip, the obtained probabilities value from this 2-class RVM is used to judge if this music clip belongs to happiness emotion or not.

## 3. Extraction of Acoustical Feature and First Level Decision Value Vector Feature

In the 2-level hierarchical sparse kernel machines, the first-level SVMs use acoustical features, while the second-level RVM adopts first-level decision vector. For acoustical features, the proposed system extracts RMS energy, tempo, chromagram, MFCCs, spectrum centroid, spectrum spread, and RSS. The extraction of these acoustical features as well as first-level decision vectors are described in the following.

### 3.1. Extraction of Acoustical Feature

#### 3.1.1. RMS Energy

RMS energy is also called root mean square energy. It computes the global energy of input signal *x* [11]. The operation is defined as follows:

(1) $$\begin{array}{c} X_{\text{RMS}}=\sqrt{\frac{1}{n}\sum_{i=1}^{n}x_{i}^{2}}, \end{array}$$

where *n* means signal's length in hundredth of a second by default.

#### 3.1.2. Tempo

Many tempo estimation methods have been proposed. The estimation of tempo is based on detecting periodicities in a range of BPMs [12]. Firstly, significant onset events are detected in the frequency domain [11]. Then find the events that best represents the tempo of the song, which means to choose the maximum periodicity score for each frame separately.

#### 3.1.3. Chromagram

Chroma which is also called harmonic pitch class profile has a strong relationship with the structure of music [13]. Chromagram is a joint distribution of signal strength over the variables of time and chroma. Chroma is a frame-based representation of audio and is similar to short time Fourier transform. In music clips, frequency components belonging to the same pitch class are extracted by chromagram and transformed to a 12-dimensional representation, including C, C#, D, D#, E, F, F#, G, G#, A, A#, and B. The chromagram can present the distribution of energy along the pitches or pitch classes [11, 14]. In [14], chromagram is defined as the remapping of time-frequency distribution. The chromagram is extracted by

(2) $$\begin{array}{c} v\left(t,k\right)=\sum_{n\in S_{k}}\frac{X_{t}\left(n\right)}{Q_{k}}\;k\in \left\{0,1,2,\ldots ,11\right\}, \end{array}$$

where *X*_*t*_(*n*) means the logarithmic magnitude of discrete Fourier transform of the *t*th frame, and *Q*_*k*_ is the number of elements in a subset of the discrete frequency space for each pitch class [15].

**Figure 2:**
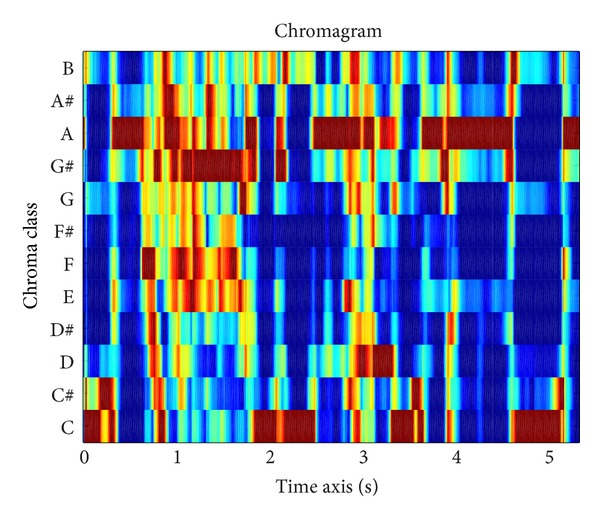
Example of chromagram from a piece of music.

In Figure 2, the chromagram from a piece of music is exemplified.

#### 3.1.4. Mel-Frequency Cepstral Coefficients (MFCCs)

After signal is digitized, a large amount of information is not needed and cost plenty of storage space. Power spectrum is often adopted to encode the signal to solve the problem [16]. It is noted that MFCCs performs similar to human auditory perception system. The feature is adopted in various research topics, including speaker recognition, speech recognition, and music emotion recognition. For example, Cooper and Foote extracted MFCCs from music signal, and they found that MFCCs are similar to music timbre expression [17]. In [18], MFCCs were also proven to be having good performance in music recommendation.

MFCCs extraction is based on spectrum. The spectrum can be extracted by using discrete Fourier transform:

(3) $$\begin{array}{c} x_{w}\left(f\right)=\sum_{n=0}^{N}x_{w}\left(n\right)\exp\left\{-\frac{2\pi fn}{N}\right\}. \end{array}$$

**Figure 3:**
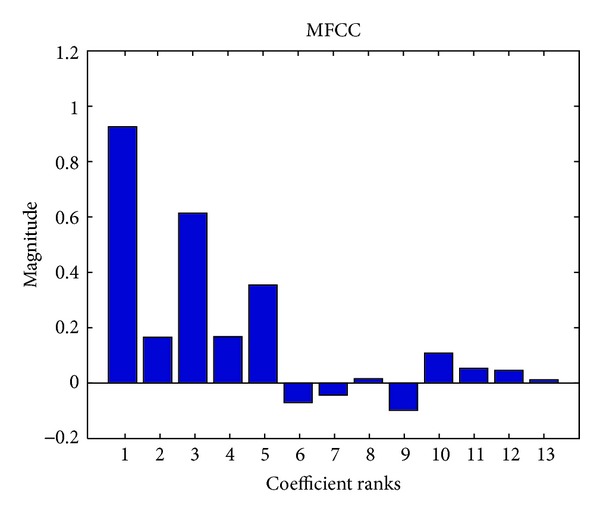
Example of average MFCCs values from a piece of music.

After power spectrum is extracted, subband energies can be extracted by using Mel filter banks and then evaluate logarithm value of the energies as follows:

(4) $$\begin{array}{c} S_{i}=\log\sum_{f=F_{l}}^{F_{h}}L\left(i,f\right){\left|X_{w}(f)\right|}^{2}, \end{array}$$

where *F*_*h*_ is the discrete frequency index corresponding to the high cutoff frequency, *F*_*l*_ is the discrete frequency index corresponding to low cutoff frequency, and *L*(*i*, *f*) is the amplitude of the *f*th discrete frequency index of the *i*th Mel window. The number of the Mel windows often ranges from 20 to 24. Finally, MFCCs is obtained by performing discrete cosine transform (DCT) [19]. In Figure 3, the average MFCCs values from a piece of music are exemplified.

#### 3.1.5. Spectrum Centroid

**Figure 4:**
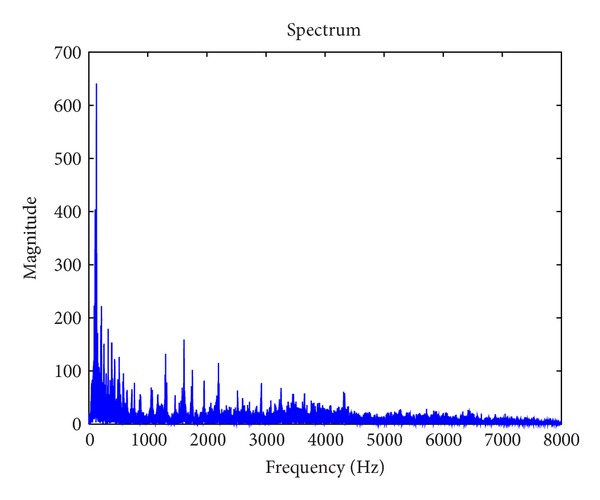
Example of a spectrum and its spectrum centroid from a frame in a piece of music.

Spectrum centroid is an economical description of the shape of the power spectrum [20–22]. Additionally, it is correlated with a major perceptual dimension of timbre, that is, sharpness.  Figure 4 gives an example of a spectrum and its spectrum centroid obtained from a frame in a piece of music. The spectrum centroid value is 2638 Hz in this example.

#### 3.1.6. Spectrum Spread

**Figure 5:**
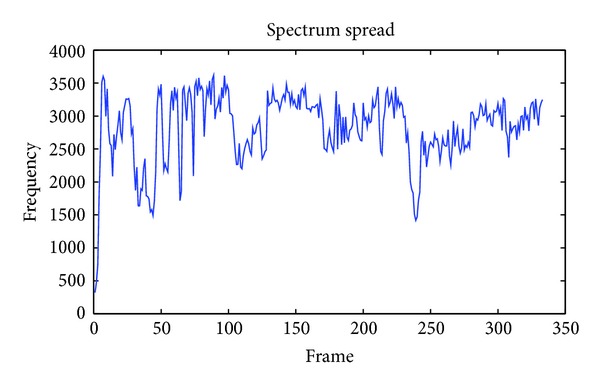
Example of spectrum spread from a piece of music.

Spectrum spread is an economical descriptor of the shape of the power spectrum that indicates whether it is concentrated in the vicinity of its centroid or else spread out over the spectrum [20–22]. It allows differentiating between tone-like and noise-like sounds. In Figure 5, an example of spectrum spread from a piece of music is provided.

#### 3.1.7. Ratio of a Spectral Flatness Measure to a Spectral Center (RSS)

RSS was proposed by Vapnik for speaker-independent emotional speech recognition [23]. RSS is the ratio of spectrum flatness to spectrum centroid and is calculated by

(5) $$\begin{array}{c} \text{RSS}=\frac{1000\times \text{SF}}{\text{SC}}, \end{array}$$

where SF denotes spectrum flatness and SC represents spectrum centroid.

### 3.2. Extraction of First-Level Decision Vector

The acoustical feature set is utilized to generate the first-level decision vector with each element being a significant value of an emotion. This approach is able to interpret the emotional content by providing multiple probabilistic class labels, rather than a single hard label [24]. For example, happiness emotion not only contains happiness content, but also other properties that are similar to the content of peace. The similarity to peaceful may cause a music clip to be recognized as an incorrect emotion class. In this example, the advantage of first-level decision vector representation is its ability to convey both the evidences of happiness and peaceful emotions. This paper uses the significant values of eight emotions (angry, happy, sad, relaxed, pleased, bored, nervous, and peaceful) to construct an emotion profile feature vector. To calculate the significant value of an emotion, we construct its 2-class SVM with calm emotion as the background side of the SVM.

## 4. Principle Component Analysis

PCA is an important mathematic technology in feature extraction approach. In this paper, PCA is implemented to reduce the dimensions of the extracted features. The first step of PCA is to calculate the *d*-dimension mean vector **u** and *d* × *d* covariance matrix Σ of the samples [25]. After that, the eigenvectors and eigenvalues are computed. Finally, the largest *k* eigenvectors are selected to form a *d* × *k* matrix *M* whose columns consist of the *k* eigenvectors. In fact, the other dimensions are noise. The PCA transformed data can be in the form

(6) $$\begin{array}{c} x^{\prime }=M^{T}\left(x-u\right). \end{array}$$

## 5. Emotion Classifier 

The emotion classifier used in the proposed system adopts a 2-level hierarchical structure of sparse kernel machines. The first-level SVMs use probability product kernel, while the second-level RVM adopts traditional radial basis function kernel with first-level decision vector feature.

### 5.1. Support Vector Machine

The SVM theory is an effective statistical technique and has drawn much attention on audio classification tasks [7]. An SVM is a binary classifier that creates an optimal hyperplane to classify input samples. This optimal hyperplane linearly divides the two classes with the largest margin [23]. Denote *T* = {(**x**_*i*_, *y*_*i*_), *i* = 1,2, …, *N*} as a training set for SVM; each pair (**x**_*i*_, *y*_*i*_) means training sample **x**_*i*_ belongs to a class *y*_*i*_, where *y*_*i*_ ∈ {+1, −1}. The fundamental concept is to choose a hyperplane which can classify **T** accurately while maximizing the distance between the two classes. This means to find a pair (**w**, *b*) such that

(7) $$\begin{array}{c} y_{i}\left(w\cdot x_{i}+b\right)>0,\;i=1,\ldots ,N, \end{array}$$

where **w** ∈ *R* ^*N*^ is normalized by itself and *b* ∈ *R*.

The pair (**w**, *b*) defines a separating hyperplane of equation

(8) $$\begin{array}{c} w\cdot x+b=0. \end{array}$$

If there exists a hyperplane satisfying (7), the set *T* is said to be linearly separable and we can change **w** and *b* so that

(9) $$\begin{array}{c} y_{i}\left(w\cdot x_{i}+b\right)>1,\;i=1,\ldots ,N. \end{array}$$

According to (9), we can derive an objective function under constraint

(10) min⁡||w||2subject  to yi(w·xi+b)>1, i=1,…,N.

Since ||**w**||^2^ is convex, we can solve (9) by applying the classical method of Lagrange multipliers:

(11) $$\begin{array}{c} \min{||w||}^{2}+\mu_{i}\left[y_{i}\left(w\cdot x_{i}+b\right)-1\right],\;i=1,\ldots ,N. \end{array}$$

We denote **U** = (*μ*_1_, *μ*_2_,…, *μ*_*N*_) as the *N* nonnegative Lagrange multipliers associated with (10). After solving (11), the optimal hyperplane has the following expansion:

(12) $$\begin{array}{c} \bar{w}=\sum_{i=1}^{N}\mu_{i}y_{i}x_{i}. \end{array}$$

$\bar{b}$ can be determined from **U** and from the Kühn-Tucker conditions. Consider

(13) μi(yi(w¯·xi+b¯)−1)=0, i=1,2,…,N.

Accordingly (11), the expected hyperplane is a linear combination of training samples. The corresponding training samples (**x**_*i*_, *y*_*i*_) with nonzero Lagrange multipliers are called support vectors. Finally, the decision value from a new data point **x** can be written as

(14) $$\begin{array}{c} \text{dec}\left(x\right)=\sum_{i=1}^{N}\mu_{i}y_{i}x_{i}\cdot x+\bar{b}. \end{array}$$

Functions that satisfy Mercer's theorem can be used as kernels. In this paper, probability product kernel is adopted.

### 5.2. Probability Product Support Vector Machine

A function can be considered as kernel function if the function satisfies Mercer's theorem. Using Mercer's theory, we can introduce a mapping function *ϕ*(**x**), such that *k*(**x**_*j*_, **x**_*i*_) = *ϕ*(**x**_*j*_)*ϕ*(**x**_*i*_). This provides the ability of handling nonlinear data, by mapping the original input space **R** ^*d*^ into some other space.

In this paper, the probability product kernel is utilized. The probability product kernel is a method of measuring similarity between distributions, and it has the property of simple and intuitively compelling conception [26]. Probability product kernel computes a generalized inner product between two probability distributions in the Hilbert space. A positive definite kernel *k* : *O* × *O* → ℝ on input space *O* and examples **o**_1_, **o**_2_,…, **o**_*m*_ ∈ *O* are defined. Firstly, the input data *x* is mapped to a probability distribution *p*(*x* | *O*), which fits separate probabilistic models *p*_1_(*x*), *p*_2_(*x*),…, *p*_*m*_(*x*) to **o**_1_, **o**_2_,…, **o**_*m*_. After that, a novel kernel *k* ^prob^(*p*_*i*_, *p*_*j*_) between probability distributions on *O* is defined. At last, a kernel between examples is needed to be defined, and the kernel is equal to *k* ^prob^ between the corresponding distributions. Consider

(15) $$\begin{array}{c} k\left(o_{i},o_{j}\right)=k^{\text{prob}}\left(p_{i},p_{j}\right). \end{array}$$

Finally, this kernel is applied to SVM and proceeded as usual. The probability product kernel between distributions *p*_*i*_ and *p*_*j*_ is defined as

(16) $$\begin{array}{c} k\left(p_{i},p_{j}\right)=\int_{x}p_{i}^{\rho }\left(x\right)p_{j}^{\rho }\left(x\right)dx={\langle p_{i}^{\rho },p_{j}^{\rho }\rangle }_{L_{2}}, \end{array}$$

where *p*_*i*_ and *p*_*j*_ are probability distributions on a space *O*. Assume that *p*_*i*_^*ρ*^, *p*_*j*_^*ρ*^ ∈ *L*_2_(*O*). *L*_2_ is a Hilbert space and *ρ* is a positive constant. Probability product kernel allows us to introduce prior knowledge of data. In this paper, we assume a *d*-dimensional Gaussian distribution of our data.

### 5.3. First-Level Decision Vector Extraction

First-level decision vector presents perception probability of each of the eight emotion-specific decisions, which is extracted from input data by collecting decision values from each model. The decision value of SVM represents the degree of similarity between model and testing data. The advantage of similarity measure can be used to find out which model fits the data most accurately [24]. Using the first-level decision vector, the most probably perceived emotion in music can be detected.

### 5.4. Relevance Vector Machine

RVM is a development of SVM. Different from SVM, RVM tries to find a considerable number of weights which has highest sparsity [27]. The model defines a conditional distribution for target class *y* = {0,1}, given an input set {**x**_1_,…, **x**_*n*_} [28]. Assume that a training data can be a linear combination of weighted nonlinear basis functions *ϕ*_*i*_(**x**), which is transformed by a logistic sigmoid function as follows:

(17) $$\begin{array}{c} f\left(x;w\right)=w^{T}\phi \left(x\right), \end{array}$$

where **w** = (*w*_1_, *w*_2_,…, *w*_*G*_), *ϕ*(**x**) = (*ϕ*_1_(**x**),*ϕ*_2_(**x**),…,*ϕ*_*G*_(**x**))^*T*^ denotes the weights. In order to make weight sparse, the Bayesian probabilistic framework is implemented to find the distribution over the weights instead of using pointwise estimation; therefore, a separate hyperparameter *a* for each of the weight parameters *w* is introduced. According to Bayes rule, the posterior probability of *w* is

(18) $$\begin{array}{c} p\left(w\;|\;y,a\right)=\frac{p\left(y\;|\;w,a\right)p\left(w\;|\;a\right)}{p\left(y\;|\;a\right)}, \end{array}$$

where *p*(*y* | **w**, **a**) is likelihood, *p*(**w** | **a**) is prior conditioned on weights **a** = [*a*_1_,…,*a*_*n*_]^*T*^, and *p*(*y* | **a**) denotes the evidence. For the reason that *y* is a binary variable, the likelihood function can be given by

(19) $$\begin{array}{c} p\left(y\;|\;w,a\right)=\prod_{i=1}^{n}{\left[\sigma \left(f\left(x_{i};w\right)\right)\right]}^{y_{i}}{\left[1-\sigma \left(f\left(x_{i};w\right)\right)\right]}^{1-y_{i}}, \end{array}$$

where *σ*(*f*) = 1/(1 + *e* ^−*f*^) is the logistic sigmoid link function. According to (18), it can be found that a significant proportion of hyperparameters tend to be infinity, and the corresponding posterior distributions of weight parameters are concentrated at zero. Therefore, the basis functions that multiplied by these parameters will not be taken for reference when training the model. As a result, the model will be sparse.

## 6. Experimental Results

In the experiments, we collected one hundred songs from two websites to construct a music emotion database. These websites are All Music Guide [29] and Last.fm [30]. As mentioned before, music may contain multiple emotions. If we know which emotion class a song most likely belongs to, we may know the main emotion of the song. Songs in Last.fm are tagged by many people on the Internet. We choose the emotion which is tagged by most people to be the ground truth of data.

**Figure 6:**
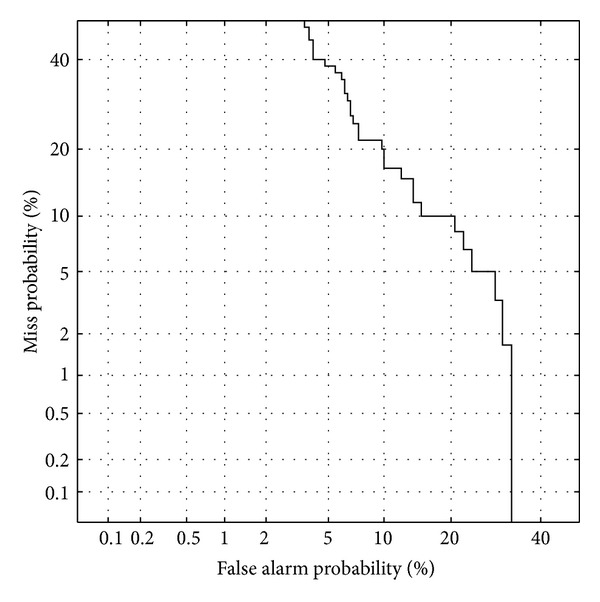
DET curve of the proposed system.

The database consists of nine classes of emotions, including happy, angry, sad, bored, nervous, relaxed, pleased, calm, and peaceful. Calm is taken as a model's opposite site when training models. Each emotion class contains twenty songs. Each song is thirty seconds long and is divided into five-second clips. Half of the songs are used as training data, and the others are used as testing data. In this paper, 240 music clips are tested. All of songs are western music and are encoded in 16 KHz WAV format. The used acoustical feature set are listed in Table 1. The whole feature set dimension is 30. The used SVM is based on LIBSVM library [31], and the used RVM is based on PTR toolbox [32]. The system performance is evaluated in terms of DET curve.  Figure 6 depicts DET curve of the proposed happiness verification system. The proposed system can achieve 13.33% equal error rate (EER). From our results, we see that the system performs well on happiness emotion verification in music.

## 7. Conclusion

Detecting emotion in music has become the concern of many researchers in recent years. In this paper, we proposed a first-level decision-vector-based music happiness emotion detection system. The proposed system adopts a hierarchical structure of sparse kernel machines. First, eight SVM models are trained based on acoustical features with probability product kernel. Then eight decision values can be extracted to construct the first-level decision vector feature. After that, these eight decision values are considered as new feature to train and test a 2-class RVM with happiness as the target side. The probability value of the RVM is used to verify happiness content in music. Experimental results reveal that the proposed system can achieve 13.33% equal error rate (EER).

## Figures and Tables

**Table 1:** The proposed acoustical feature set.

Feature class	Feature name (dimension of feature)
Dynamic	RMS energy (1)
Rhythm	Tempo (1)
Timbre	MFCCs (13), spectrum centroid (1), spectrum spread (1), RSS (1)
Tonality	Chromagram (12)
